# Characterizing the tuberculosis and type 2 diabetes mellitus comorbidity in a South African cohort using untargeted GCxGC-TOFMS metabolomics

**DOI:** 10.1007/s11306-025-02389-y

**Published:** 2026-01-19

**Authors:** Karla Reinecke, Léanie Kleynhans, Katharina Ronacher, Du Toit Loots

**Affiliations:** 1 Biomedical and Molecular Metabolism Research (BioMMet), Department of Biochemistry, Faculty of Natural and Agricultural Sciences, North-West University, Potchefstroom, South Africa; 2 DSI-NRF Centre of Excellence for Biomedical Tuberculosis Research, South African Medical Research Council Centre for Tuberculosis Research, Division of Molecular Biology and Human Genetics, Department of Biomedical Sciences, Stellenbosch University, Cape Town, South Africa; 3 Mater Research Institute - The University of Queensland, Translational Research Institute, Brisbane, Australia; 4 Australian Infectious Diseases Research Centre, The University of Queensland, Brisbane, Australia

**Keywords:** Tuberculosis, Diabetes, Comorbidity, Metabolomics, Urine

## Abstract

**Introduction:**

Tuberculosis (TB) and type 2 diabetes mellitus (T2DM) are highly prevalent diseases resulting in high mortality rates globally. Furthermore, T2DM increases susceptibility to TB and vice versa, worsening disease outcomes. This comorbidity is, however, not well described nor understood, despite its rising prevalence globally.

**Objectives:**

This investigation aimed to better characterize the urinary metabolic profiles of patients with the TB and T2DM comorbidity in a South African cohort, to better understand its metabolic basis and associated clinical implications.

**Methods:**

Using untargeted GCxGC-TOFMS metabolomics, urine samples from 17 patients with TB and T2DM and 34 healthy controls were analyzed and statistically compared to identify significantly altered urinary metabolites.

**Results:**

TB-T2DM comorbid patients were characterized by altered metabolism of: (1) tryptophan and kynurenine (reduced kynurenic acid, anthranilic acid, picolinic acid) associated with changes to NAD^+^ synthesis and a redox imbalance, (2) nucleotides (reduced 3-aminoisobutyric acid, orotic acid, thymine, β-alanine, adenine, hypoxanthine), (3) tyrosine (reduced 3,4-dihydroxyphenylglycol, 4-hydroxy-3-methoxyphenylglycol, hydroxyphenylpyruvate), (4) lipids (reduced dec-2-enedioate, adipic acid, methylmalonic acid), (5) reduced concentrations of various glycine conjugates associated with glycine depletion, and (6) reduced urinary concentrations of various gut microbial metabolites indicative of microbial dysbiosis.

**Conclusion:**

These results indicate several metabolic disruptions to amino acids, nucleotides, lipids, NAD⁺ homeostasis and the host microbiome, in TB-T2DM patients, mainly driven by inflammation and oxidative stress. Overall, the findings indicate synergistic amplification of metabolic stress, associated with immune suppression and TB-T2DM disease progression, and subsequently suggests how TB increases T2DM susceptibility and vice versa, as foundation for further investigations.

**Supplementary Information:**

The online version contains supplementary material available at 10.1007/s11306-025-02389-y.

## Introduction

Tuberculosis (TB), caused by *Mycobacterium tuberculosis* (*M.tb*), is a major global epidemic, with approximately 10.8 million new cases and a resulting 1.25 million deaths, reported for 2023 (World Health Organization, 2024). Nearly a quarter of the global population is infected, making TB the leading cause of death from a single infectious agent worldwide (World Health Organization, 2024). Transmission occurs via inhalation of aerosolized droplets with *M.tb* reaching alveoli and triggering an immune response, resulting in *M.tb* internalization by resident macrophages, followed by granuloma formation to limit bacterial spread (Leung, 1999; Philips & Ernst, 2012). The infection may be eradicated or remain latent, depending on host’s immune competence (Alsayed & Gunosewoyo, 2023; Behr et al., 2021; Lin & Flynn, 2010). Immunocompromised individuals risk progression to active TB, presenting with cough, fever, and weight loss (Acharya et al., 2020; Leung, 1999), and risk latent TB reactivation (5% risk within two years following infection) (Menzies et al., 2018). Additional risk factors for contracting the disease include the occurrence of immunosuppressive diseases such as diabetes mellitus (DM) and acquired immunodeficiency syndrome (AIDS) (World Health Organization, 2024).

On the other hand, DM affects approximately 589 million adults globally and resulted in 3.4 million deaths in 2024 globally (International Diabetes Federation, 2025). With 4.3 million cases (Diabetes Alliance, 2023), South Africa is the African country with the highest prevalence. DM, defined by chronic hyperglycemia (HbA1c ≥ 6.5%), is classified into type 1 (T1DM), which is characterized by autoimmune β-cell destruction and reduction in insulin secretion (Knip & Siljander, 2008) and type 2 (T2DM), that is associated with insulin resistance developed due to poor lifestyle and obesity, leading to pancreatic damage and reduced insulin secretion (Banday et al., 2021; Wang et al., 2020). T2DM accounts for approximately 90% of all DM cases (Zhang et al., 2014) and is therefore the focus of this study.

T2DM impairs the immune system and consequently increases the risk of contracting active TB disease by approximately three-fold (Niazi & Kalra, 2012; Sane Schepisi et al., 2018). Consequently, Workneh et al. (2017) reported approximately 16% of TB patients present with T2DM. T2DM also increases the likelihood for TB relapses after treatment, lower cure rates and increased morbidity (Adane et al., 2023; Habib et al., 2024; Lee et al., 2014). Furthermore, TB-DM comorbid patients also have a 3.8-fold increased risk of developing multiple drug-resistant TB (Evangelista et al., 2020). Anti-T2DM treatment also affects TB disease outcomes. Chen et al. (2020) reported that high doses of dipeptidyl peptidase-4 (DPP4) inhibitors (> 20 average defined daily doses) were associated with a 2.19-fold increased risk of TB. In contrast, subsequent large-cohort study by Wang et al. (2023) found no association between DPP4 inhibitors and TB risk, consistent with the findings of a systematic review and meta-analysis by Grenet et al. (2021), which demonstrated no link between DPP4 inhibitor use and respiratory infections risk. However, Chen et al. (2025) observed that the use of DPP4 inhibitors considerably lowered the risk of developing pulmonary TB in T2DM patients. Conversely, clinical studies consistently demonstrate that metformin reduces TB incidence (Lin et al., 2020; Meregildo-Rodriguez et al., 2022; Zhang & He, 2020). On the other hand, *M.tb* infection induces stress-associated hyperglycemia via pro-inflammatory cytokines and reactive oxygen species (ROS) production (Jeon & Murray, 2008; Niazi & Kalra, 2012; Shastri et al., 2018; Workneh et al., 2017; Yorke et al., 2017), which stimulate hepatic glucose release (Sharma et al., 2019) and contribute to metabolic dysregulation (Magee et al., 2018). A metabolomics study done by Du Preez and Loots (2013), showed a 10 fold increase in the concentrations of the norepinephrine derivative; normetanephrine, in TB-positive patients, explaining the associated glucose intolerance (additionally contributing to the increased D-gluconic acid d-lactone detected in their study), since elevated levels of normetanephrine are also associated with insulin resistance and impaired insulin secretion (Murabayashi et al., 2018). Anti-TB drugs also interact with T2DM medication. Rifampicin lowers plasma levels of biguanides and sulphonylureas (Niazi & Kalra, 2012), while isoniazid antagonizes sulphonylureas (Dartois & Rubin, 2022; Yorke et al., 2017).

Metabolomics serves as a useful tool to better understand TB-T2DM, since T2DM is a metabolic disease (Li et al., 2009; Yen et al., 2023) and TB also results in severe metabolic changes (Du Preez & Loots, 2013; Isa et al., 2018; Luies and Loots, 2016; Vrieling et al., 2019). To date, as far as we are aware, only one such metabolomics study has been done describing the metabolic changes associated with TB-T2DM, using patient collected plasma, reporting and briefly describing reduced choline, citrulline, histidine, ornithine, and tryptophan in TB-T2DM patients when compared with healthy controls (Vrieling et al., 2019). Plasma metabolomics reflects the metabolic state of an individual at the exact time of sample collection (González-Domínguez et al., 2020), while urinary metabolomics on the other hand, captures metabolic fluctuations over time (Dunn & Ellis, 2005) and is also considered easier to collect and prepare due to its low protein content (Du Preez & Loots, 2013; Khamis et al., 2015; Zhang et al., 2012). Therefore, this study employed untargeted GCxGC-TOFMS urinary metabolomics to compare TB-T2DM patients to healthy controls with and without latent TB, to comprehensively characterize the metabolic profiles of TB-T2DM patients in a South African cohort.

## Methods and materials

### Participants

Voluntarily participating study participants (*n* = 125; *n* = 97 healthy controls (HC) with and without latent TB and *n* = 28 TB-T2DM patients) were recruited from hospitals and community clinics situated in the Western Cape, South Africa. The participant cohort was refined into a final cohort (*n* = 51; *n* = 34 HC with and without latent TB and *n* = 17 TB-T2DM) by application of various inclusion and exclusion criteria. The HC included individuals accompanying patients to the hospitals and clinics with (*n* = 15) and without (*n* = 19) latent TB. Latent TB status was confirmed using the QuantiFERON-Gold In-Tube assay (Qiagen, cat #622536) and the specific cutoff values outlined in the manufacturers’ instructions. A principal component analysis (PCA) was performed on the HC group to determine whether latent TB influenced metabolic profiles. As no statistical differentiation was observed between participants with and without latent TB (results not shown), these subgroups were combined into a single HC group (*n* = 34) to increase statistical power. Moreover, individuals were excluded from the HC group if they had any acute respiratory tract infection in the 4 weeks prior to recruitment, suffered from chronic hyperglycemia, were previously or currently diagnosed with T2DM, tested positive for active TB disease by a GeneXpert assay and/or sputum culture and smear or were suffering from any severe systemic condition. Participants were included in the TB-T2DM group if they were either newly diagnosed with pulmonary TB, or recurrent TB with TB treatment completed at least 2 months prior to recruitment, had previously been diagnosed with T2DM or had an HbA1c ≥ 6.5% (excluding gestational or steroid-induced diabetes) with- and without T2DM treatment. The TB diagnosis was confirmed by two separate positive sputum smears and/or a positive mycobacteria growth indicator tube culture, and/or positive polymerase chain reaction (PCR) for the presence of *M.tb*. Study participants were generally excluded if diagnosed with any alternative medical conditions (chronic bronchitis/emphysema/asthma, cancer, HIV positive), received steroid therapy within 6 months of recruitment, participated in any drug or vaccine trial, were pregnant, abused alcohol (> 3 alcoholic beverages per day) or illicit drugs and had no permanent address. Table 1 shows the biographical information of the study participants. Ethics approval for the larger scope of this study has been obtained from the Health Research Ethics Committee (HREC) of Stellenbosch University (reference number: N13/05/064; project ID: 4095). The current study (Ethics number: NWU-00096-23-A1-02) falls under a larger study at the North-West University with the title: “The characterization of tuberculosis-diabetes mellitus co-morbidity in a South African cohort using metabolomics” for which ethics approval has been obtained (Ethics number: NWU-00096-23-A1).

**Table 1:** Sociodemographic characteristics of healthy control and tuberculosis-type 2 diabetes comorbid participants

	HC with latent TB (*n* = 19)	HC without latent TB (*n* = 15)	TB-T2DM (*n* = 17)
Age (average ± standard deviation)	41.21 ± 10.02	32.60 ± 10.45	47.35 ± 9.45
Sex (% female/% male)	53/47	73/27	47/53
Smoking (%)	74	80	65
HbA1c (% average ± standard deviation)	5.42 ± 0.44	5.34 ± 0.43	9.49 ± 2.20
**Patients on T2DM treatments: (%)**			
No treatment	–	–	47.1
Only insulin	–	–	5.9
Only metformin	–	–	17.6
Metformin with other anti-T2DM drugs	–	–	23.5
Insulin and metformin	–	–	5.9
**Duration of T2DM: (%)**			
Less than 1 year	–	–	29.4
1–5 years	–	–	17.6
6–15 years	–	–	29.4
Undocumented	–	–	23.5

HC, healthy control; TB, tuberculosis; TB-T2DM, tuberculosis-type 2 diabetes mellitus; HbA1c, glycated hemoglobin; T2DM, type 2 diabetes mellitus

### Urine sampling and storage

Study participants were asked to provide a urine sample in standard urine pots provided by the study nurses or clinicians. The samples were initially stored at -80 °C at Stellenbosch University, after which they were transported to North-West University and stored at -80 °C until the commencement of the GCxGC-TOFMS metabolomic analysis.

### Reagents and chemicals

The following reagents were used: 3-phenylbutyric acid (internal standard), methoxyamine hydrochloride (MOX-HCl) in pyridine and N, O-bis(trimethylsilyl)trifuoroacetamide (BSTFA) with 1% trimethylsilyl chloride (TMCS) and acetonitrile from Burdick and Jackson brand (Honeywell International Inc., Muskegon, USA).

### Sample Preparation

Equal amounts (20 µL) of all patient urine samples were used to compile a pooled quality control (QC) sample, from which aliquots were prepared to be extracted and analyzed with each sample batch (samples were randomly assigned to batches). Following creatinine normalization to 1 µmol, the corresponding urine volume was combined with 100 µL of internal standard solution (3-phenyl butyric acid, 50 ppm) and 300 µL of ice-cold acetonitrile, vortexed, incubated on ice for 10 min and centrifuged at 10 000 g for 10 min. The supernatant was then transferred to a 2 mL GC vial and dried under nitrogen at 40 °C. Derivatization involved: (1) methoximation with 50 µL MOX-HCl (15 mg/mL) in pyridine at 60 °C for 60 min, and (2) trimethylsilylation with 50 µL BSTFA-TMCS, at 60 °C for 60 min. The final sample was transferred to a glass insert, placed in the GC vial and capped.

### GCxGC-TOFMS analysis and processing

Prepared samples were randomly selected and analyzed (alongside QC samples, extraction blank samples, system suitability test samples comprising of fatty acid methyl esters (FAMEs), placed intermittently throughout each batch) using the Pegasus 4D GC×GC-TOFMS (Leco Africa (Pty) Ltd, Johannesburg, South Africa) equipped with an Agilent 7890 GC. A 1:3 split ratio was employed to inject 1 µl of each sample with the front inlet temperature at 270 °C. Purified helium served as a carrier gas with a constant flow of 3 mL/min. First-dimensional chromatographical separation was achieved with a Restek Rxi-5Sil MS primary column (28.2 m; 250 μm internal diameter and 0.25 μm film thickness), with the primary oven ramping from 70 °C (2 min hold) to 300 °C (2 min hold) at 5 °C/min. Second-dimensional separation was achieved by a Restek Rxi-17 capillary column (1.32 m × 250 μm diameter × 0.25 μm film thickness), with the secondary oven ramping from 85 °C (2 min hold) to 310 °C (4.5 min hold) at 5.5 °C/min. The modulator was programmed to ramp from 100 °C (2 min hold) to 310 °C (12 min hold) at 5 °C/min, with 0.5 s cold/hot nitrogen pulses every 3 s. A 350 s acquisition delay excluded solvent detection. Transfer line and ion source were set to 270 °C and 200 °C, respectively, with − 70 eV filament bias and 150 V detector voltage. Mass spectra were acquired over 50–950 m/z at 200 spectra per second. Data was processed using Leco Corporation ChromaTOF software (v4.32) with peak identification based on 70% spectral library match, signal-to-noise ratio of 200 and minimum of 3 apex peaks. Furthermore, the Statistical compare function was used for peak alignment based on similarity in mass spectra and retention times.

### Data management

Data clean-up was performed using Microsoft Excel prior to statistical analysis. Relative concentrations (mmol/mol creatinine) were calculated by normalization to the internal standard, 3-phenylbutyric acid. A 50% filter was applied to retain only compounds present in at least half of at least one of the two experimental groups. Batch correction (using quantile equating) and a coefficient of variation (CV) filter (retaining compounds with CV ≤ 50%) were applied using QC samples. Zero values detected for a compound were replaced with half the smallest detected value to reflect the lower detection limit (Luies and Loots, 2016). MetaboAnalyst 6.0, based on the statistical program, “R” (v4.3.2), was employed for data normalization with log transformation and autoscaling, as well as further statistical analysis. Principal component analysis (PCA) was performed on the final data set to determine if any natural separation between the HC and TB-T2DM groups exists. Biomarker selection was based on a multi-statistical approach using: (1) PLS-DA (VIP > 1), (2) t-test (*p* < 0.1), and (3) effect size (Cohen’s d > 0.8) (Du Preez & Loots, 2013).

## Results

**Fig. 1:**
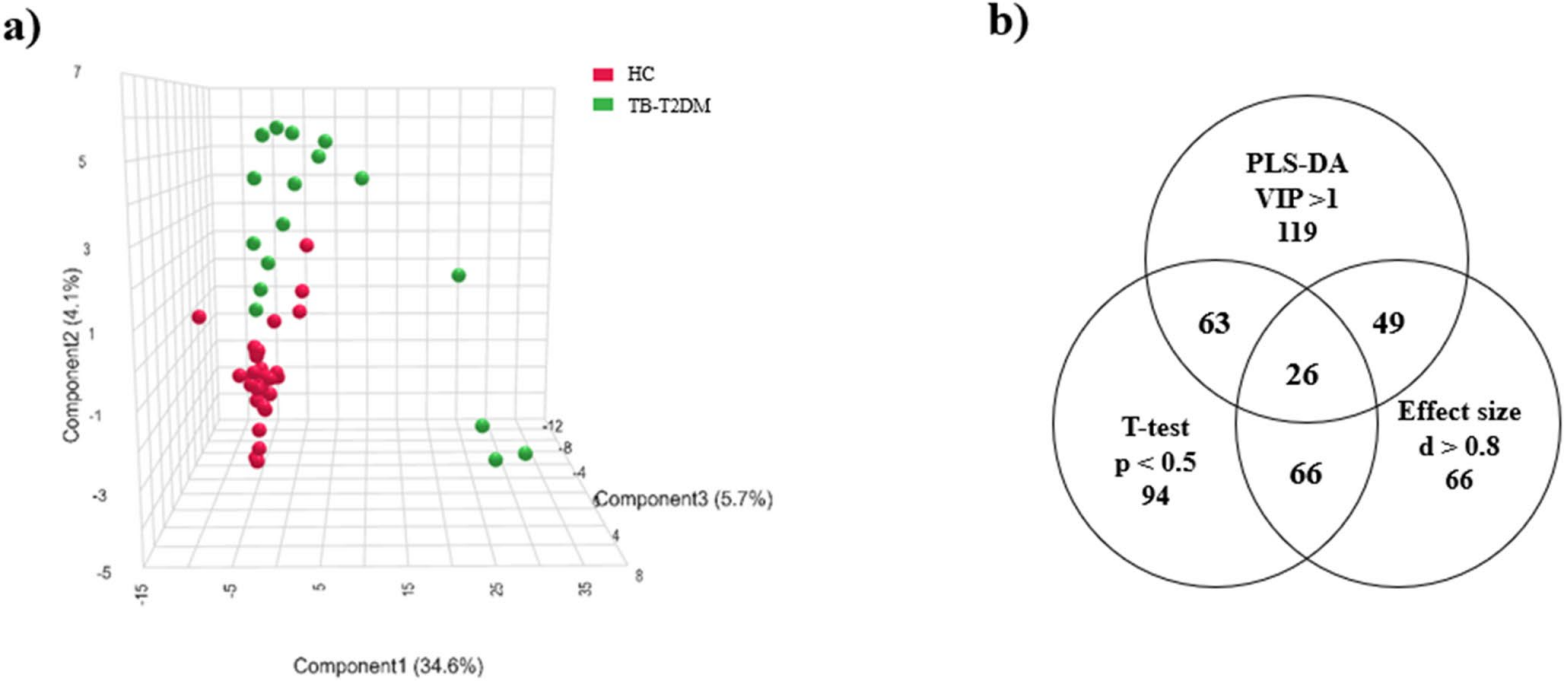
**a** PLS-DA plot of the TB-T2DM and healthy control group showing group separation. **b** Venn diagram illustrating the number of metabolites that met each criterium as well as the overlap of common metabolites, forming the list of biomarkers

Following data processing, cleaning, mass spectral deconvolution, peak identification and alignment, 2161 urinary metabolites were detected. Removal of unidentified compounds yielded a final data matrix of 280 metabolites. The PCA performed using all patient samples and all 280 identified metabolites, showed no natural separation between TB-T2DM and HC groups (S1 Fig. 1a), likely due to demographical variation (age, sex, diet, treatment, etc.), introducing metabolic “noise”. Outliers may have arisen from this “noise”, preventing clear discrimination between the two experimental groups by PCA. The available clinical information did not explain the definite origin of the noise, though multiple factors may contribute to it. Therefore, a multi-statistical approach was employed for metabolite marker selection using: (1) PLS-DA (VIP > 1), (2) t-test (*p* < 0.5), and (3) fold change (log2 (FC) > |2|) (S1 Fig. 1b). The 19 metabolites that satisfied all three criteria were included in a “noise-reduced” dataset. PCA of this noise-reduced dataset showed separation between the TB-T2DM and HC groups (S1 Fig. 1c). Random forest analysis identified 4 outlier samples only from the HC group, which were removed from the dataset (Wu & Wang, 2008).

A PLS-DA model (Fig. 1a) was constructed and evaluated by 10-fold cross-validation repeated 10 times. The model achieved an accuracy of 84.1 ± 1.8% with a predictive ability of Q² = 0.513 and R^2^ = 0.975 (Szymańska et al., 2012). Variability observed within the TB-T2DM group likely reflects differences in T2DM treatment and duration as reported in Table 1. These patients were still included to preserve cohort size, enhancing statistical power and avoiding the introduction of a degree of bias. Despite treatment, all TB-T2DM patients still presented with HbA1c > 6.5%, confirming persistent hyperglycemia and uncontrolled T2DM. Finally, a 2nd Venn diagram (Fig. 1b) was constructed to illustrate the number of metabolites meeting each criterion and the overlap of common metabolites between each test. Only compounds satisfying all three criteria: VIP > 1 (PLS-DA, Fig. 1b), *p* < 0.05 (t-test), and d > 0.8 (effect size), were included in the final list of biomarkers, listed in Table 2.

**Table 2:** Metabolite markers of the TB-T2DM comorbidity and the healthy controls

Compound (ChemSpider ID)	PLS-DA	t-test	Effect size	Average relative concentration (mmol/mol creatinine); Standard deviation	
	VIP	*p*-value	Cohen’s d	Healthy control	TB-T2DM
**Tryptophan metabolism/kynurenine pathway/NAD** ^**+**^					
Kynurenic acid (3712)	1.124	0.023	0.915	0.158 ; 0.38	0.038 ; 0.045
Anthranilic acid (222)	1.030	0.013	0.979	0.283 ; 0.559	0.157 ; 0.261
Picolinic acid (993)	1.075	0.023	0.851	0.533 ; 1.331	0.183 ; 0.155
**Amino acid metabolism**					
2-Methylcrotonyl glycine (4945715)	1.048	0.018	0.892	1.710 ; 2.929	0.617 ; 0.756
N-(2-methyl-1-oxobutyl) glycine (168243)	1.042	0.016	0.929	0.071 ; 0.172	0.022 ; 0.034
Isobutyryl glycine (9030891)	1.116	0.005	1.137	0.215 ; 0.352	0.069 ; 0.102
**Tyrosine metabolism**					
3,4-Dihydroxyphenylglycol (82648)	2.476	0.001	1.583	0.15 ; 0.302	0.003 ; 0.01
4-Hydroxy-3-methoxyphenylglycol (10348)	1.088	0.027	0.816	1.409 ; 1.684	0.791 ; 0.79
Hydroxyphenylpyruvate (954)	1.044	0.018	0.906	1.094 ; 1.996	0.309 ; 0.275
**Pyrimidine metabolism**					
Orotic acid (942)	1.052	0.024	0.907	0.593 ; 1.319	0.131 ; 0.170
3-Aminoisobutyric acid (58481)	1.044	0.029	0.857	4.676 ; 17.452	2.174 ; 5.453
β-Alanine (234)	1.060	0.041	0.806	0.156 ; 0.514	0.020 ; 0.026
Thymine (1103)	2.097	< 0.001	1.674	0.155 ; 0.391	0.010 ; 0.022
**Purine metabolism**					
Adenine (185)	1.046	0.028	0.879	0.278 ; 0.761	0.105 ; 0.159
Hypoxanthine (768)	1.033	0.029	0.862	2.385 ; 5.655	1.139 ; 1.627
**Dicarboxylic / lipid metabolism**					
Dec-2-enedioate (21237627)	1.912	< 0.001	1.589	0.025 ; 0.041	0.003 ; 0.003
Adipic acid (191)	1.045	0.018	0.897	1.66 ; 5.102	0.282 ; 0.396
Methylmalonic acid (473)	2.269	0.018	1.079	0.623 ; 2.642	0.002 ; 0.006
**Gut microflora metabolism**					
Phenylacetylglutamine (83292)	3.027	< 0.001	2.111	0.672 ; 2.019	0.124 ; 0.315
Indoxyl (45861)	1.087	0.028	0.808	0.525 ; 1.871	0.149 ; 0.169
3-(3-Hydroxyphenyl) propanoic acid (89)	1.049	0.023	0.849	0.400 ; 0.984	0.096 ; 0.089
Cyclohexylamine (7677)	1.081	0.023	0.844	12.039 ; 41.082	2.638 ; 2.347
Syringol (6774)	1.808	0.001	1.414	0.729 ; 3.471	0.028 ; 0.073
Syringic acid (10289)	1.070	0.016	0.983	32.022 ; 101.646	7.425 ; 10.644
2,6-Dihydroxybenzoic acid (8974)	1.364	0.003	1.266	0.027 ; 0.047	0.004 ; 0.006
**Nicotine consumption**					
Trans-3’-hydroxycotinine (97080)	1.067	0.026	0.926	0.182 ; 0.499	0.035 ; 0.048

## Discussion

**Fig. 2:**
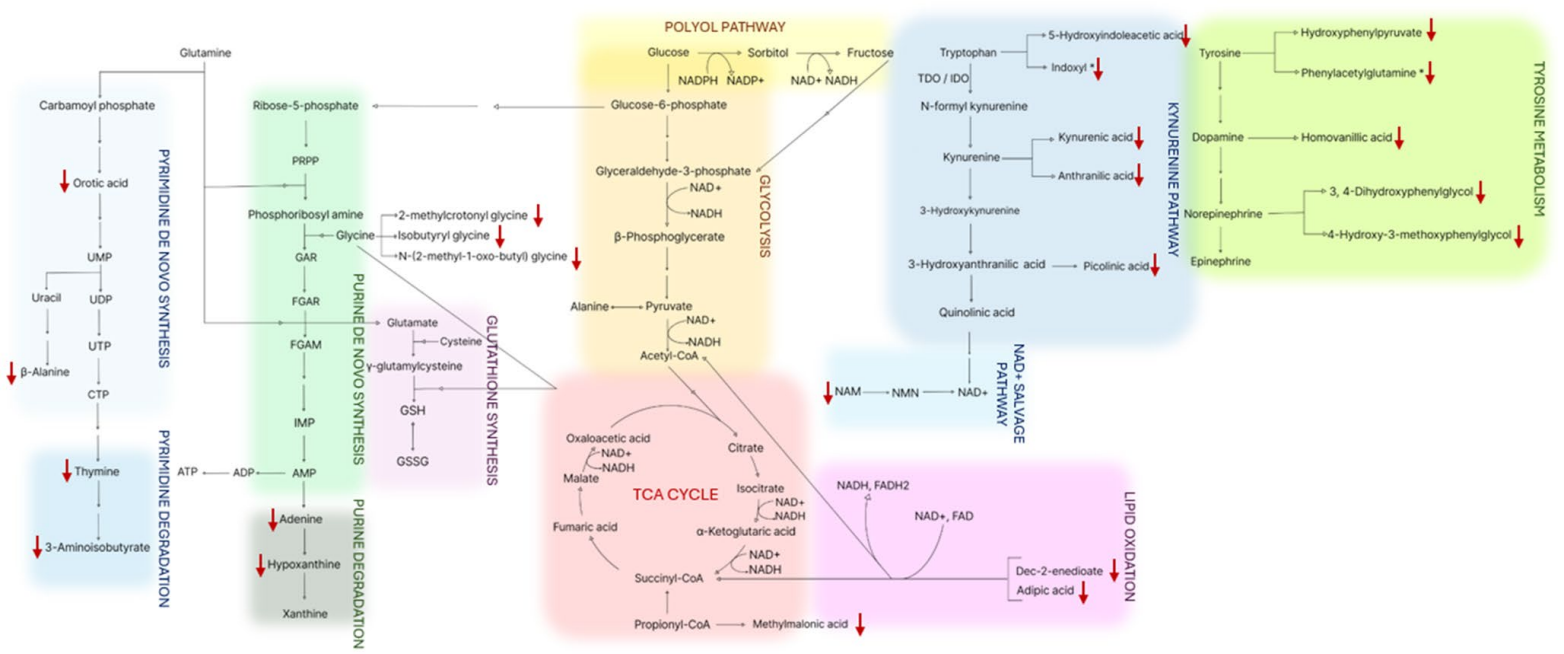
A schematic summary of the metabolic changes associated with the tuberculosis and type 2 diabetes mellitus observed in this investigation. The directional change in the urinary concentrations of the metabolites indicated to be reduced (↓), relative to the healthy control group. UMP, uridine monophosphate; UDP, uridine diphosphate; UTP, uridine triphosphate; CTP, cytidine triphosphate; PRPP, phosphoribosyl pyrophosphate; GAR, Glycine amide ribonucleotide; FGAR, Formyl glycinamide ribonucleotide; IMP, inosine monophosphate; AMP, adenosine monophosphate; ADP, adenosine diphosphate; ATP, adenosine triphosphate; GSH, glutathione; GSSG, glutathione disulfide; NAD^+^, nicotinamide adenine dinucleotide; NADH, reduced nicotinamide adenine dinucleotide; NAM, nicotinamide NMN, nicotinamide mononucleotide; TDO, tryptophan-2,3-dioxygenase; IDO, indoleamine-2,3-dioxygenase. *Metabolites associated with microbial metabolism and not human metabolism

Table 2 lists the selected metabolite markers that most significantly differ between the urinary metabolic profiles of the TB-T2DM patients and HC. The selected metabolite markers indicate perturbations in multiple metabolic pathways including the metabolism of tryptophan, glycine, tyrosine, nucleotides, dicarboxylic acids/lipids and gut microbiome. The perturbations in these metabolic pathways are discussed in the following sections and are illustrated in Fig. 2.

### Tryptophan metabolism, kynurenine pathway and NAD+

The kynurenine pathway is a major route of tryptophan catabolism in mammals (Mandi & Vecsei, 2012; Martin et al., 2020). Tryptophan is converted to kynurenine by the dioxygenases, tryptophan 2,3-dioxygenase (TDO) and indoleamine 2,3-dioxygenase-1 (IDO-1), along with kynurenine formamidase (Badawy, 2017; Martin et al., 2020) as seen in Fig. 2. TDO primarily acts in the liver, while IDO-1 functions in extrahepatic tissues, particularly immune cells (Badawy, 2017; Pires et al., 2020). IDO-1 catalyzes the rate-limiting step of this pathway and is induced by pro-inflammatory cytokines interferon-γ (IFN-γ) and tumor necrosis factor-α (TNF-α) (González et al., 2008; Mandi & Vecsei, 2012). These inflammatory mediators are secreted by airway epithelial cells, dendritic cells, alveolar macrophages, type II pneumocytes, and CD4^+^/CD8^+^ T cells, promoting macrophage activation during pulmonary *M. tb* infection (Etna et al., 2014; Sharma et al., 2007). Elevated IDO-1 activity and higher kynurenine/tryptophan ratios have been associated with an increased mortality in pulmonary TB, suggesting its potential prognostic use (Suzuki et al., 2012). Similarly, in T2DM, hyperglycemia has also been associated with increased TNF-α (Navarro-González & Mora-Fernández, 2008; Wang et al., 2020), further contributing to IDO-1 activation. IDO-1 activation shifts tryptophan catabolism toward kynurenine pathway in immune cells as a negative feedback mechanism to modulate inflammation (Mandi & Vecsei, 2012; Martin et al., 2020). The kynurenine pathway metabolites have also been shown to impair the immune response by inhibiting CD4⁺ T-cell activity, contributing to immunosuppression (Singhal & Cheng, 2018).

Although not selected as a metabolite marker in this study using the strict selection criteria, the tryptophan catabolite, 5-hydroxyindoleacetic acid (Luies and Loots, 2016) was also significantly reduced in TB-T2DM urine samples of this study (0.357 vs. 0.993 mmol/mol creatinine, *p* = 0.009), aligning with Vrieling et al. (2019) reporting lower plasma tryptophan concentrations in TB-T2DM patients compared to healthy controls. The concentrations of kynurenic acid, anthranilic acid and picolinic acid were furthermore significantly reduced in TB-T2DM patients when compared to HC in this study. Although these metabolites are associated with the kynurenine pathway (Badawy, 2017; Martin et al., 2020), their reductions indicate a flux towards elevated NAD^+^-synthesis (Badawy, 2017; Mandi & Vecsei, 2012; Martin et al., 2020) as illustrated in Fig. 2, as a means to correct for the typically disrupted NAD^+^/NADH ratio associated with both TB and diabetes (Ido et al., 1997; Pajuelo et al., 2018; Sun et al., 2015; Williamson et al., 1993). Further confirmation of this are the reduced urinary concentrations of nicotinamide (NAM) (0.062 vs. 0.167 mmol/mol creatinine, *p* = 0.037), though not selected as a metabolic marker using the strict selection criteria. In a salvage pathway, nicotinamide phosphoribosyl transferase (NAMPT), a rate-limiting enzyme, catalyzes the conversion of NAM to nicotinamide mononucleotide (NMN) (Gallí et al., 2010; Singhal & Cheng, 2018), illustrated in Fig. 2. Activated immune cells exhibit upregulated gene expression of NAMPT (Gallí et al., 2010). This upregulation has also been observed in vascular endothelial cells, alveolar epithelial cells, inflammatory cells and other cells in individuals with acute lung injury and pulmonary inflammation (Zhang et al., 2011). The release of pro-inflammatory cytokines, including IL-1β, which has also been seen to be elevated in TB-T2DM patients, has been implicated in the upregulation of NAMPT, which in turn, results in elevated IL-8 secretion by pulmonary A549 cells (Ronacher et al., 2015; Zhang et al., 2011). The final step in NAD⁺ biosynthesis is the conversion of NMN to NAD⁺ (Fukushima & Lopaschuk, 2016). The aforementioned imbalanced NAD⁺/NADH ratio, affects fatty acid, glucose, TCA cycle metabolism, energy production (Xie et al., 2020), DNA repair (Hou et al., 2018; Wilk et al., 2020), and oxidative stress (Sultana et al., 2016; Wang et al., 2014), and is associated with T2DM progression (Sultana et al., 2016; Williamson et al., 1993) and fatty liver disease (Akie et al., 2015). The latter is not only strongly associated with T2DM (Kumar et al., 2022), but also TB (Amarapurkar & Ghansar, 2007), and hence the susceptibility of TB patients for developing T2DM.

Kynurenic acid also functions as noncompetitive N-methyl-D-aspartate receptor (NMDAR) antagonist (Mandi & Vecsei, 2012). In mice, NMDAR overactivation led to insulin resistance and hyperlipidemia, while its inhibition reversed these effects (Huang et al., 2021). Furthermore, Huang et al. (2021) proposed that activation of pancreatic NMDARs initiates a cascade of events resulting in mitochondria-mediated apoptosis of the pancreatic β-cells: firstly, the increase in the intracellular Ca²⁺ concentration depolarizes mitochondrial membrane potential, impairment of mitochondrial oxidative phosphorylation, increase in oxidative stress/ROS production and ultimately, reduction in pancreatic insulin secretion. Thus, the observed reduction in kynurenic acid concentration, would activate NMDAR (Asp et al., 2011), contributing to reduced insulin secretion (Huang et al., 2017) that naturally results in the T2DM progression (Peterson & Shulman, 2018).

In summary, the upregulation of NAD⁺ biosynthesis via the kynurenine pathway in the TB-T2DM patients, likely represents an attempt to correct the redox balance by restoring the disrupted NAD⁺/NADH ratio, which is perturbed by several pathophysiological mechanisms associated with both TB and T2DM (Singhal & Cheng, 2018). Poor glycemic control (associated to T2DM as well as TB) promotes the upregulation of glycolysis, fatty acid oxidation, the TCA cycle (in insulin-independent tissues) and the polyol pathway – resulting in a decreased NAD⁺/NADH ratio (Garg & Gupta, 2022). Furthermore, oxidative stress induced by inflammation associated with both TB and DM (Amaral et al., 2021; Pasupuleti et al., 2020), damages mitochondrial proteins, impairing mitochondrial function and disrupting NAD⁺ recycling via the electron transport chain (Zhao et al., 2021). The decreased NAD⁺/NADH ratio furthermore, impairs the immune response mediated mainly via TNF by sirtuins, NAD^+^ dependent deacetylases (Gallí et al., 2010), a likely mechanism by which diabetes increases the susceptibility for TB infection.

### Oxidative stress and Glycine metabolism

Hyperglycemia promotes ROS production via glucose auto-oxidation, glycation of antioxidant enzymes, and non-enzymatic glycation reactions, all of which impair anti-oxidative mechanisms (Kaneto et al., 2010; Pasupuleti et al., 2020). In insulin-independent cells, excess intracellular glucose surpasses cellular glycolytic ability and enters alternative pathways that further elevate ROS (Li et al., 2008). In both T2DM and TB, increased lipolysis and fatty acid oxidation contribute to mitochondrial ROS generation, particularly via electron leakage at complexes I and III of the OXPHOS system (Las et al., 2020; Li et al., 2008). Furthermore, a TB metabolomics study done by Du Preez and Loots (2013), showed elevated glucose oxidation which results in the production of H_2_O_2_ in TB. Pancreatic β-cells, due to their low antioxidant capacity, are especially vulnerable to oxidative damage and ROS-induced damage induces β-cell apoptosis, impairing insulin secretion (Las et al., 2020; Wang et al., 2020).

Vrieling et al. (2019) reported significantly reduced plasma glycine in TB-T2DM individuals compared to healthy controls. This is substantiated by observed reduction in the concentrations of glycine conjugates in this study. Glycine is essential for synthesizing glutathione (GSH), the most abundant intracellular antioxidant (Lu, 2013). GSH synthesis begins with ligation of glutamate and cysteine by glutamate-cysteine ligase (GCL), composed of catalytic (GCLC) and modifier (GCLM) subunits (Lu, 2013). GCLM mediates feedback inhibition of GCL by GSH to regulate levels, and GSH synthetase then adds glycine to form GSH (Fig. 2) (Lu, 2013). In T2DM, reduced glycine and GSH levels (Sekhar et al., 2011) reflect impaired antioxidant capacity and elevated oxidative stress. GSH mitigates oxidative stress by reducing oxidative species and forming glutathione disulfide (GSSG) (Butkowski & Jelinek, 2016). In TB-T2DM, elevated ROS accelerates GSH oxidation to GSSG, resulting in feedback inhibition on GCL and promoting GSH synthesis from glycine, cysteine, and glutamate (Lu, 2013). This increased GSH demand likely contributes to glycine depletion previously observed in TB-T2DM patients (Vrieling et al., 2019).

In this study, the aforementioned reduced glycine conjugates: 2-methylcrotonyl glycine, N-(2-methyl-1-oxobutyl) glycine and isobutyryl glycine in the TB-T2DM patients, confirms the previously observed glycine reduction. 2-Methylcrotonyl glycine is formed by conjugating glycine with 2-methylcrotonyl-CoA, an intermediate in isoleucine degradation (Fontaine et al., 1996), while, N-(2-methyl-1-oxobutyl) glycine and isobutyryl glycine are derived from 2-methylbutanoyl-CoA and isobutyryl-CoA respectively, metabolites involved in catabolism of branched-chain amino acids (BCAAs), isoleucine, valine, and leucine (Guda et al., 2007; Shibata & Sakamoto, 2016). T2DM is reportedly associated with elevated urinary BCAA concentrations (Siddik & Shin, 2019; Theron et al., 2024), suggesting the reduction in their degradation, and our results also confirming the latter.

### Tyrosine metabolism

Norepinephrine (NE) is synthesized from tyrosine via dopamine (DA) (Fig. 2) and binds postjunctional receptors to mediate various physiological responses (Alaniz et al., 1999). Excess NE is either reabsorbed or metabolized (Linares et al., 1987). TB has been associated with increased NE secretion (Du Preez & Loots, 2013). In this study, urinary 3,4-dihydroxyphenylglycol and 4-hydroxy-3-methoxyphenylglycol, two NE catabolites (Denfeld et al., 2018; Peaston & Weinkove, 2004), were significantly reduced in TB-T2DM patients, suggesting increased NE receptor binding and utilization. Homovanillic acid, a DA catabolite (Irwin et al., 1988), though not selected as a biomarker, was also markedly decreased (0.565 vs. 1.087 mmol/mol creatinine, *p* = 0.028), supporting enhanced DA utilization. Additionally, reduced concentrations of hydroxyphenylpyruvate, an intermediate in tyrosine catabolism (Gertsman et al., 2015; Irwin et al., 1988), further indicate altered tyrosine metabolism in the TB-T2DM group. This suggests a mechanism by which TB patients are more susceptible to developing diabetes and diabetes progression/severity (Du Preez & Loots, 2013).

### Nucleotide metabolism

Glutamine is crucial for de novo purine and pyrimidine synthesis, as seen in Fig. 2 (Parveen & Bishai, 2024). *M.tb* reprograms host glutamine metabolism toward energy production via glutaminolysis, depleting glutamine and impairing nucleotide de novo synthesis (Koeken et al., 2019; Parveen & Bishai, 2024). To compensate, nucleotide salvage pathways are upregulated to preserve nucleotide availability (Chandel, 2021). The TB-T2DM patients in this study showed significantly reduced orotic acid, an intermediate in the pyrimidine de novo synthesis pathway (Chandel, 2021), likely due to glutamine depletion (Koeken et al., 2019). The decreased concentrations of other pyrimidine catabolites in this study; 3-aminoisobutyric acid, thymine and β-alanine (Chandel, 2021), support enhanced nucleotide salvage activity.

Similarly, the purine degradation products; adenine and hypoxanthine (Chandel, 2021), were also reduced in TB-T2DM, indicative of impaired de novo purine synthesis due to aforementioned glutamine and glycine depletion, and compensatory purine salvage pathway activation (Fig. 2) (Ducati et al., 2011). Given that TB-T2DM exhibits reduced glycolysis and TCA flux in insulin-dependent cells as well as a compromised mitochondrial function, salvage pathways, requiring less ATP than de novo synthesis (Villela et al., 2011), are preferentially employed (Chandel, 2021), explaining the reduced purine catabolite excretion. Purine metabolism also supports cyclic adenosine monophosphate (cAMP) synthesis from adenine, essential for insulin secretion (Carvalho et al., 2018). Considering this, the compromised nucleotide synthesis induced by TB contributes to susceptibility of these patients to getting T2DM and would further contribute to diabetes progression/severity.

### Dicarboxylic acid/lipid metabolism

In T2DM, production various TCA cycle intermediates are impaired in insulin-dependent cells (Peterson & Shulman, 2018). To compensate, dicarboxylic acids like dec-2-enedioate and adipic acid may be oxidized to generate acetyl-CoA and succinyl-CoA, supplementing the TCA cycle, as seen in Fig. 2 (Mingrone et al., 2013). Methylmalonic acid, derived from propionyl-CoA (Chalmers & Lawson, 1982), also supplements TCA cycle via succinyl-CoA (Peterson & Shulman, 2018). The significantly reduced urinary concentrations of these metabolites in TB-T2DM patients supports their increased metabolic utilization to sustain energy production. Moreover, their enhanced oxidation may also elevate oxidative stress, potentially exacerbating hyperinsulinemia (Las et al., 2020b).

### Gut microbial metabolism

TB is associated with altered gut microbial composition and reduced microbial diversity (Liu et al., 2021). TB is reported to reduce microbial populations of *Bifidobacteria*, *Lactobacillus* and *Bacteroides* (Hu et al., 2019; Negi et al., 2020). T2DM, additionally, is associated with reductions in both oral and gut microbiota (Shillitoe et al., 2012). Furthermore, diabetic enteropathy, a T2DM complication, further alters the gastrointestinal (GI) tract, causing diarrhea, constipation, and abdominal discomfort (Zhong et al., 2016).

The decreased urinary excretion of the observed microbial metabolites in TB-T2DM patients in this study further supports the presence of the above-mentioned gut dysbiosis. Phenylacetylglutamine and indoxyl are derived from microbial metabolism of tyrosine and tryptophan, respectively (Barrios et al., 2016). 3-(3-Hydroxyphenyl) propanoic acid results from microbial breakdown of procyanidins (Ou et al., 2014) and cyclohexylamine from cyclamate (Drasar et al., 1972). Syringol and syringic acid originate from microbial fermentation of lignin and anthocyanins respectively (Hidalgo et al., 2012; Ohra-aho et al., 2016), while unabsorbed aspirin may be converted to 2,6-dihydroxybenzoic acid by intestinal microbes (Sankaranarayanan et al., 2020). The overall reduction of these urinary microbial metabolites supports decreased microbial diversity and activity due to TB- and T2DM-associated gut microbiota disturbances.

### Nicotine consumption

Trans-3’-hydroxycotinine, a nicotine catabolite (Bergen et al., 2015), was significantly reduced. Information regarding participant smoking habits indicated less smoking in TB-T2DM patients. Moreover, TB-related pulmonary symptoms such as persistent coughing and chest discomfort (Storla et al., 2008), may have discouraged nicotine use, and patients are also advised to stop smoking, explaining the decline.

## Conclusion

This investigation provides a characterization of the urinary metabolic profile of the TB-T2DM comorbidity using untargeted GC×GC-TOFMS metabolomics in a South African cohort. The findings show that metabolic disturbances associated with TB-T2DM are driven by inflammatory responses and oxidative stress with substantial downstream effects on amino acid metabolism, NAD^+^/NADH balance, nucleotide biosynthesis and lipid oxidation. This investigation observed the activation of the kynurenine pathway, likely mediated by release of the pro-inflammatory cytokines IFN-γ and TNF-α in response to *M.tb* infection, resulting in the activation of NMDARs. This elevates mitochondrial ROS production and causes pancreatic dysfunction, due to limited antioxidants present in pancreatic β-cells, impairing insulin secretion, thereby facilitating T2DM pathogenesis in TB and the TB-T2DM patients. Additionally, the hyperglycemia seen in T2DM, depletes cellular NAD⁺ pools due to the reduction in mitochondrial NAD^+^ recycling due to mitochondrial damage caused by ROS, resulting in a compensatory upregulation of NAD^+^ synthesis via the kynurenine pathway and elevated glycolysis, fatty acid oxidation, the TCA cycle in insulin-independent tissues and the polyol pathway. This NAD^+^ depletion and compensatory synthesis upregulation is further substantiated by the reduction in NAM excretion, from which NAD^+^ is synthesized. A compromised NAD⁺/NADH ratio weakens host immunity, particularly TNF-driven responses mediated by NAD^+^-dependent sirtuins, thereby increasing susceptibility to TB infection or reactivation in T2DM patients. The elevated oxidative stress is further substantiated by reduced urinary excretion of glycine conjugates, suggesting increased glutathione synthesis in response to ROS. Several detected metabolites in this investigation points to a disruption in nucleotide metabolism in TB-T2DM patients. This study shows that the TB-associated glutamine depletion impairs de novo purine and pyrimidine synthesis, initiating a shift to the salvage pathways. This is reflected in reduced concentrations of nucleotide degradation products in urine. Furthermore, this investigation identified significant perturbations in the gut microbiome of TB-T2DM patients, indicative of a reduction in microbial diversity.

The strict inclusion and exclusion criteria employed in this metabolomic investigation resulted in a relatively small cohort, increasing the susceptibility of the findings to variability associated with factors such as age, sex, and treatment protocols, all of which can influence urinary metabolic profiles. Nonetheless, the findings of this study support previous hypotheses regarding the mechanisms by which TB patients have an increased susceptibility to developing diabetes and vice versa, and highlights the disease mechanisms in each which would have a compounding effect towards and increase disease severity.

## Supplementary Information

Below is the link to the electronic supplementary material.

Supplementary Material 1

## Data Availability

The raw data, supporting the findings of this study, can be acquired from the author upon reasonable request.

## Abbreviations

DM: Diabetes mellitus
GCxGC-TOFMS: Two-dimensional gas chromatography combined with time-of-flight mass spectrometry
HC: Healthy control group
IDO-1: Indole amine 2,3-dioxygenase-1
IFN-γ: Interferon-γ
M.tb: Mycobacterium tuberculosis
NAD^+^: Nicotinamide adenine dinucleotide
NAM: Nicotinamide
ROS: Reactive oxygen species
T2DM: Type 2 diabetes mellitus
TB: Tuberculosis
TB-T2DM: Tuberculosis-type 2 diabetes mellitus
TNF-α: Tumor necrosis factor-α

## References

[CR1] Acharya, B., Acharya, A., Gautam, S., Ghimire, S. P., Mishra, G., Parajuli, N., & Sapkota, B. (2020). Advances in diagnosis of tuberculosis: An update into molecular diagnosis of Mycobacterium tuberculosis. *Molecular Biology Reports*, *47*, 4065–4075. 10.1007/s11033-020-05413-7 32248381 10.1007/s11033-020-05413-7

[CR2] Adane, H. T., Howe, R. C., Wassie, L., & Magee, M. J. (2023). Diabetes mellitus is associated with an increased risk of unsuccessful treatment outcomes among drug-susceptible tuberculosis patients in ethiopia: A prospective health facility-based study. *J Clin Tuberc Other Mycobact Dis*, *31*, 100368. 10.1016/j.jctube.2023.100368 37122611 10.1016/j.jctube.2023.100368 PMC10130346

[CR3] Akie, T. E., Liu, L., Nam, M., Lei, S., & Cooper, M. P. (2015). OXPHOS-Mediated induction of NAD + Promotes complete oxidation of fatty acids and interdicts Non-Alcoholic fatty liver disease. *PLOS ONE*, *10*, e0125617. 10.1371/journal.pone.0125617 25933096 10.1371/journal.pone.0125617 PMC4416931

[CR4] Alaniz, R. C., Thomas, S. A., Perez-Melgosa, M., Mueller, K., Farr, A. G., Palmiter, R. D., & Wilson, C. B. (1999). Dopamine beta-hydroxylase deficiency impairs cellular immunity. *Proceedings of the National Academy of Sciences of the United States of America*, *96*, 2274–2278. 10.1073/pnas.96.5.2274 10051631 10.1073/pnas.96.5.2274 PMC26773

[CR5] Alsayed, S. S. R., & Gunosewoyo, H. (2023). Tuberculosis: Pathogenesis, current treatment regimens and new drug targets. *International Journal of Molecular Sciences*. 10.3390/ijms24065202 10.3390/ijms24065202 PMC10049048 36982277

[CR6] Amaral, E. P., Vinhaes, C. L., Oliveira-de-Souza, D., Nogueira, B., Akrami, K. M., & Andrade, B. B. (2021). The interplay between systemic Inflammation, oxidative Stress, and tissue remodeling in tuberculosis. *Antioxidants and Redox Signaling*, *34*, 471–485. 10.1089/ars.2020.8124 32559410 10.1089/ars.2020.8124 PMC8020551

[CR7] Amarapurkar, A., & Ghansar, T. (2007). Fatty liver: Experience from Western India. *Annals of Hepatology*, *6*, 37–40. 10.1016/S1665-2681(19)31951-9 17297427

[CR8] Asp, L., Johansson, A. S., Mann, A., Owe-Larsson, B., Urbanska, E. M., Kocki, T., Kegel, M., Engberg, G., Lundkvist, G. B. S., & Karlsson, H. (2011). Effects of pro-inflammatory cytokines on expression of kynurenine pathway enzymes in human dermal fibroblasts. *Journal of Inflammation*. 10.1186/1476-9255-8-25 10.1186/1476-9255-8-25 PMC3204223 21982155

[CR9] Badawy, A. A. B. (2017). Tryptophan availability for kynurenine pathway metabolism across the life span: Control mechanisms and focus on aging, exercise, diet and nutritional supplements. *Neuropharmacology*, *112*, 248–263. 10.1016/j.neuropharm.2015.11.015 26617070 10.1016/j.neuropharm.2015.11.015

[CR10] Banday, M. Z., Sameer, A. S., & Nissar, S. (2021). Pathophysiology of diabetes: An overview. *Avicenna Journal of Medicine*, *10*, 174–188. 10.4103/ajm.ajm_53_20 10.4103/ajm.ajm_53_20 PMC7791288 33437689

[CR11] Barrios, C., Spector, T. D., & Menni, C. (2016). Blood, urine and faecal metabolite profiles in the study of adult renal disease. *Archives of Biochemistry and Biophysics*, *589*, 81–92. 10.1016/j.abb.2015.10.006 26476344 10.1016/j.abb.2015.10.006

[CR12] Behr, M. A., Kaufmann, E., Duffin, J., Edelstein, P. H., & Ramakrishnan, L. (2021). Latent tuberculosis: Two centuries of confusion. *American Journal of Respiratory and Critical Care Medicine*, *204*, 142–148. 10.1164/rccm.202011-4239PP 33761302 10.1164/rccm.202011-4239PP PMC8650795

[CR13] Bergen, A. W., Michel, M., Nishita, D., Krasnow, R., Javitz, H. S., Conneely, K. N., Lessov-Schlaggar, C. N., Hops, H., Zhu, A. Z., Baurley, J. W., McClure, J. B., Hall, S. M., Baker, T. B., Conti, D. V., Benowitz, N. L., Lerman, C., Tyndale, R. F., & Swan, G. E. (2015). Drug metabolizing enzyme and transporter gene Variation, nicotine Metabolism, prospective Abstinence, and cigarette consumption. *Plos One*, *10*, e0126113. 10.1371/journal.pone.0126113 26132489 10.1371/journal.pone.0126113 PMC4488893

[CR14] Butkowski, E. G., & Jelinek, H. F. (2016). Hyperglycaemia, oxidative stress and inflammatory markers. *Redox Report*, *22*, 257–264. 10.1080/13510002.2016.1215643 28277069 10.1080/13510002.2016.1215643 PMC6837501

[CR15] Carvalho, D. S., De Almeida, A. A., Borges, A. F., & Vannucci Campos, D. (2018). Treatments for diabetes mellitus type II: New perspectives regarding the possible role of calcium and cAMP interaction. *European Journal of Pharmacology*, *830*, 9–16. 10.1016/j.ejphar.2018.04.002 29679542 10.1016/j.ejphar.2018.04.002

[CR16] Chalmers, R. A., & Lawson, A. M. (1982). Disorders of propionate and methylmalonate metabolism. *Organic Acids in Man*. 10.1007/978-94-009-5778-7_11

[CR17] Chandel, N. S. (2021). Nucleotide metabolism. *Cold Spring Harbor Perspectives in Biology*. 10.1101/cshperspect.a040592 10.1101/cshperspect.a040592 PMC8247561 34210662

[CR18] Chen, H. H., Hsieh, M. C., Ho, C. W., Chen, C. C., Hsu, S. P., Lin, C. L., & Kao, C. H. (2020). Effects of dipeptidyl peptidase-4 inhibitor treatment doses on tuberculosis in patients with diabetes: A long-term nationwide population-based cohort study. *Ann Palliat Med*, *9*, 2817–2825. 10.21037/apm-20-278 32787376 10.21037/apm-20-278

[CR19] Chen, Y. G., Wei, J. C., Yen, F. S., Sung, C. Y., Huang, Y. H., Yu, T. S., Tsai, F. J., Hwu, C. M., & Hsu, C. C. (2025). Target trial emulation of DPP-4 inhibitors in patients with T2DM for pulmonary tuberculosis: A nationwide observational data. *Bmc Medicine*, *23*, 587. 10.1186/s12916-025-04423-1 41137015 10.1186/s12916-025-04423-1 PMC12553194

[CR20] Dartois, V. A., & Rubin, E. J. (2022). Anti- tuberculosis treatment strategies and drug development: Challenges and priorities. *Nature Reviewe Microbiology*, *20*, 685–701. 10.1038/s41579-022-00731-y 10.1038/s41579-022-00731-y PMC9045034 35478222

[CR21] Denfeld, Q. E., Habecker, B. A., & Woodward, W. R. (2018). Measurement of plasma norepinephrine and 3,4-dihydroxyphenylglycol: Method development for a translational research study. *BMC Research Notes*, *11*, 248. 10.1186/s13104-018-3352-3 29673396 10.1186/s13104-018-3352-3 PMC5909231

[CR22] Diabetes Alliance. (2023). 2023 Diabetes Summit Report 2023.

[CR23] Drasar, B. S., Renwick, A. G., & Williams, R. T. (1972). The role of the gut flora in the metabolism of cyclamate. *Biochemical Journal*, *129*, 881–890. 10.1042/bj1290881 4655823 10.1042/bj1290881 PMC1174233

[CR24] Du Preez, I., & Loots, D. T. (2013). New sputum metabolite markers implicating adaptations of the host to Mycobacterium tuberculosis, and vice versa. *Tuberculosis*, *93*, 330–337. 10.1016/j.tube.2013.02.008 23477940 10.1016/j.tube.2013.02.008

[CR25] Ducati, R. G., Breda, A., & Basso, L. A. (2011). Purine salvage pathway in Mycobacterium tuberculosis. *Current Medicinal Chemistry*, *18*, 1258–1275. 10.2174/092986711795029627 21366536 10.2174/092986711795029627

[CR26] Dunn, W. B., & Ellis, D. I. (2005). Metabolomics: Current analytical platforms and methodologies. *TrAC Trends in Analytical Chemistry*, *24*, 285–294. 10.1016/j.trac.2004.11.021

[CR27] Etna, M. P., Giacomini, E., Severa, M., & Coccia, E. M. (2014). Pro- and anti-inflammatory cytokines in tuberculosis: A two-edged sword in TB pathogenesis. *Seminars in Immunology*, *26*, 543–551. 10.1016/j.smim.2014.09.011 25453229 10.1016/j.smim.2014.09.011

[CR28] Evangelista, M., Maia, R., Toledo, J. P., Abreu, R. G., & Barreira, D. (2020). Tuberculosis associated with diabetes mellitus by age group in brazil: A retrospective cohort study, 2007–2014. *The Brazilian Journal of Infectious Diseases*, *24*, 130–136. 10.1016/j.bjid.2020.03.005 32298639 10.1016/j.bjid.2020.03.005 PMC9392016

[CR29] Fontaine, M., Briand, G., Ser, N., Armelin, I., Rolland, M. O., Degand, P., & Vamec, J. (1996). Metabolic studies in twin brothers with 2-methylacetoacetyl-CoA thiolase deficiency. *Clinica Chimica Acta*, *255*, 67–83. 10.1016/0009-8981(96)06391-7 10.1016/0009-8981(96)06391-7 8930414

[CR30] Fukushima, A., & Lopaschuk, G. D. (2016). Acetylation control of cardiac fatty acid β-oxidation and energy metabolism in obesity, diabetes, and heart failure. *Biochimica Et Biophysica Acta*, *1862*, 2211–2220. 10.1016/j.bbadis.2016.07.020 27479696 10.1016/j.bbadis.2016.07.020

[CR31] Gallí, M., Van Gool, F., Rongvaux, A., Andris, F., & Leo, O. (2010). The nicotinamide phosphoribosyltransferase: A molecular link between metabolism, inflammation, and cancer. *Cancer Research*. 10.1158/0008-5472.CAN-09-2465 10.1158/0008-5472.CAN-09-2465 20028851

[CR32] Garg, S. S., & Gupta, J. (2022). Polyol pathway and redox balance in diabetes. *Pharmacological Research*, *182*, 106326. 10.1016/j.phrs.2022.106326 35752357 10.1016/j.phrs.2022.106326

[CR33] Gertsman, I., Gangoiti, J. A., Nyhan, W. L., & Barshop, B. A. (2015). Perturbations of tyrosine metabolism promote the indolepyruvate pathway via Tryptophan in host and Microbiome. *Molecular Genetics and Metabolism*, *114*, 431–437. 10.1016/j.ymgme.2015.01.005 25680927 10.1016/j.ymgme.2015.01.005

[CR35] González, A., Varo, N., Alegre, E., Díaz, A., & Melero, I. (2008). Immunosuppression routed via the kynurenine pathway: A biochemical and pathophysiologic approach. *Advances in Clinical Chemistry*. 10.1016/s0065-2423(07)00007-8. 155 – 97. 10.1016/s0065-2423(07)00007-8 18429497

[CR34] González-Domínguez, R., González-Domínguez, Á., Sayago, A., & Fernández-Recamales, Á. (2020). Recommendations and best practices for standardizing the Pre-Analytical processing of blood and urine samples in metabolomics. *Metabolites*. 10.3390/metabo10060229 10.3390/metabo10060229 PMC7344701 32503183

[CR36] Grenet, G., Mekhaldi, S., Mainbourg, S., Auffret, M., Cornu, C., Cracowski, J. L., Gueyffier, F., Lega, J. C., & Cucherat, M. (2021). DPP-4 inhibitors and respiratory infection: A systematic review and Meta-analysis of the cardiovascular outcomes trials. *Diabetes Care*, *44*, e36–e37. 10.2337/dc20-2018 33436399 10.2337/dc20-2018

[CR37] Guda, P., Guda, C., & Subramaniam, S. (2007). Reconstruction of pathways associated with amino acid metabolism in human mitochondria. *Genomics Proteomics Bioinformatics*, *5*, 166–176. 10.1016/S1672-0229(08)60004-2 18267298 10.1016/S1672-0229(08)60004-2 PMC5054205

[CR38] Habib, M. A., Afrin, K., Efa, S. S., Hossain, M. D., Islam, M. R., Rahman, M. M., Islam, N., Afroz, F., Rahim, M. A., & Hossain, M. D. (2024). Effects of diabetes mellitus on retreatment of tuberculosis: A multi-centered case-control study from Bangladesh. *Journal of Clinical Tuberculosis and Other Mycobacterial Diseases*, *36*, 100450. 10.1016/j.jctube.2024.100450 38770156 10.1016/j.jctube.2024.100450 PMC11103381

[CR39] Hidalgo, M., Oruna-Concha, M. J., Kolida, S., Walton, G. E., Kallithraka, S., Spencer, J. P. E., Gibson, G. R., & Pascual-Teresa, D., S (2012). Metabolism of anthocyanins by human gut microflora and their influence on gut bacterial growth. *Journal of Agricultural and Food Chemistry*, *60*, 3882–3890. 10.1021/jf3002153 22439618 10.1021/jf3002153

[CR40] Hou, Y., Lautrup, S., Cordonnier, S., Wang, Y., Croteau, D. L., Zavala, E., Zhang, Y., Moritoh, K., O’Connell, J. F., Baptiste, B. A., Stevnsner, T. V., Mattson, M. P., & Bohr, V. A. (2018). NAD + supplementation normalizes key alzheimer’s features and DNA damage responses in a new AD mouse model with introduced DNA repair deficiency. *Proceedings of the National Academy of Sciences*, *115*, E1876–E1885. 10.1073/pnas.1718819115 10.1073/pnas.1718819115 PMC5828618 29432159

[CR41] Hu, Y., Yang, Q., Liu, B., Dong, J., Sun, L., Zhu, Y., Su, H., Yang, J., Yang, F., Chen, X., & Jin, Q. (2019). Gut microbiota associated with pulmonary tuberculosis and dysbiosis caused by anti-tuberculosis drugs. *Journal of Infection*, *78*, 317–322. 10.1016/j.jinf.2018.08.006 30107196 10.1016/j.jinf.2018.08.006

[CR43] Huang, X. T., Yue, S. J., Li, C., Huang, Y. H., Cheng, Q. M., Li, X. H., Hao, C. X., Wang, L. Z., Xu, J. P., Ji, M., Chen, C., Feng, D. D., & Luo, Z. Q. (2017). A sustained activation of pancreatic NMDARs is a novel factor of beta-Cell apoptosis and dysfunction. *Endocrinology*, *158*, 3900–3913. 10.1210/en.2017-00366 28938426 10.1210/en.2017-00366

[CR42] Huang, X. T., Yang, J. X., Wang, Z., Zhang, C. Y., Luo, Z. Q., Liu, W., & Tang, S. Y. (2021). Activation of N-methyl-D-aspartate receptor regulates insulin sensitivity and lipid metabolism. *Theranostics*, *11*, 2247–2262. 10.7150/thno.51666 33500723 10.7150/thno.51666 PMC7797674

[CR44] Ido, Y., Kilo, C., & Williamson, J. R. (1997). Cytosolic NADH/NAD+, free radicals, and vascular dysfunction in early diabetes mellitus. *Diabetologia*, *40*(Suppl 2), 115–117. 10.1007/s001250051422 10.1007/s001250051422 9248714

[CR45] International Diabetes Federation (2025). IDF Diabetes Atlas. https://diabetesatlas.org/data/en/

[CR46] Irwin, J., Kopin, J. H., White, & Bankiewicz, K. (1988). A new approach to biochemical evaluation of brain dopamine metabolism. *Cellular and Molecular Neurobiology*, *8*, 171–179. 10.1007/bf00711243 3044592 10.1007/BF00711243 PMC11567391

[CR47] Isa, F., Collins, S., Hee Lee, M., Decome, D., Dorvil, N., Joseph, P., Smith, L., Salerno, S., Wells, M. T., Fischer, S., Bean, J. M., Pape, J. W., Johnson, W. D., Fitzgerald, D. W., & Rhee, K. Y. (2018). Mass spectrometric identification of urinary biomarkers of pulmonary tuberculosis. *EBioMedecine*, *31*, 157–165. 10.1016/j.ebiom.2018.04.014 10.1016/j.ebiom.2018.04.014 PMC6013777 29752217

[CR48] Jeon, C. Y., & Murray, M. B. (2008). Diabetes mellitus increases the risk of active tuberculosis: A systematic review of 13 observational studies. *Plos Medicine*, *5*, e152. 10.1371/journal.pmed.0050152 18630984 10.1371/journal.pmed.0050152 PMC2459204

[CR49] Kaneto, H., Katakami, N., Matsuhisa, M., & Matsuoka, T. A. (2010). Role of reactive oxygen species in the progression of type 2 diabetes and atherosclerosis. *Mediators Inflammation**2010*, 453892. 10.1155/2010/453892 10.1155/2010/453892 PMC2825658 20182627

[CR50] Khamis, M. M., Adamko, D. J., & El-Aneed, A. (2015). Mass spectrometric based approaches in urine metabolomics and biomarker discovery. *Mass Spectrometry Reviews*, *36*, 115–134. 10.1002/mas.21455 25881008 10.1002/mas.21455

[CR51] Knip, M., & Siljander, H. (2008). Autoimmune mechanisms in type 1 diabetes. *Autoimmun Rev*, *7*, 550–557. 10.1016/j.autrev.2008.04.008 18625444 10.1016/j.autrev.2008.04.008

[CR52] Koeken, V. A. C. M., Lachmandas, E., Riza, A., Matzaraki, V., Li, Y., Kumar, V., Oosting, M., Joosten, L. A. B., Netea, M. G., & Van Crevel, R. (2019). Role of glutamine metabolism in host defense against Mycobacterium tuberculosis infection. *The Journal of Infectious Diseases*, *219*, 1662–1670. 10.1093/infdis/jiy709 30541099 10.1093/infdis/jiy709

[CR53] Kumar, P., Rawat, S., Kakar, A., & Sinha, A. K. (2022). Prevalence of non-alcoholic fatty liver disease among diabetes, prediabetes and healthy population. *J Family Med Prim Care*, *11*, 7640–7643. 10.4103/jfmpc.jfmpc_856_22 36994063 10.4103/jfmpc.jfmpc_856_22 PMC10041021

[CR54] Las, G., Oliveira, M. F., & Shirihai, O. S. (2020). Emerging roles of β-cell mitochondria in type-2-diabetes. *Molecular Aspects of Medicine*, *71*, 100843. 10.1016/j.mam.2019.100843 31918997 10.1016/j.mam.2019.100843

[CR56] Lee, P. H., Lin, H. C., Huang, A. S., Wei, S. H., Lai, M. S., & Lin, H. H. (2014). Diabetes and risk of tuberculosis relapse: Nationwide nested case-control study. *PLoS One*, *9*, e92623. 10.1371/journal.pone.0092623 24663327 10.1371/journal.pone.0092623 PMC3963913

[CR57] Leung, A. N. (1999). Pulmonary tuberculosis: The essentials. *Radiology*, *210*, 307–322. 10.1148/radiology.210.2.r99ja34307 10207408 10.1148/radiology.210.2.r99ja34307

[CR58] Li, N., Frigerio, F., & Maechler, P. (2008). The sensitivity of pancreatic beta-cells to mitochondrial injuries triggered by lipotoxicity and oxidative stress. *Biochemical Society Transactions*, *36*, 930–934. 10.1042/BST0360930 18793163 10.1042/BST0360930

[CR59] Li, X., Xu, Z., Lu, X., Yang, X., Yin, P., Kong, H., Yu, Y., & Xu, G. (2009). Comprehensive two-dimensional gas chromatography/time-of-flight mass spectrometry for metabonomics: Biomarker discovery for diabetes mellitus. *Analytica Chimica Acta*, *633*, 257–262. 10.1016/j.aca.2008.11.058 19166731 10.1016/j.aca.2008.11.058

[CR61] Lin, P. L., & Flynn, J. L. (2010). Understanding latent tuberculosis: A moving target. *The Journal of Immunology*, *185*, 15–22. 10.4049/jimmunol.0903856 20562268 10.4049/jimmunol.0903856 PMC3311959

[CR60] Lin, K., Luo, C. W., Chen, S. P., Tu, D. G., Lin, M. S., & Kuan, Y. H. (2020). α-Glucosidase inhibitor can effectively inhibit the risk of tuberculosis in patients with diabetes: A nested Case-Control study. *BioMed Research International*, *2020*, 1–12. 10.1155/2020/8085106 10.1155/2020/8085106 PMC7254087 32509871

[CR62] Linares, O. A., Jacquez, J. A., Zech, L. A., Smith, M. J., Sanfield, J. A., Morrow, L. A., Rosen, S. G., & Halter, J. B. (1987). Norepinephrine metabolism in humans. Kinetic analysis and model. *The Journal of Clinical Investigation*, *80*, 1332–1341. 10.1172/JCI113210 3316275 10.1172/JCI113210 PMC442388

[CR63] Liu, Y., Wang, J., & Wu, C. (2021). Microbiota and tuberculosis: A potential role of Probiotics, and postbiotics. *Frontiers in Nutrition*, *8*, 626254. 10.3389/fnut.2021.626254 34026804 10.3389/fnut.2021.626254 PMC8138307

[CR64] Lu, S. C. (2013). Glutathione synthesis. *Biochimica Et Biophysica Acta*, *1830*, 3143–3153. 10.1016/j.bbagen.2012.09.008 22995213 10.1016/j.bbagen.2012.09.008 PMC3549305

[CR65] Luies, L. and Loots, D.T. (2016) Tuberculosis metabolomics reveals adaptations of man and microbe in order to outcompete and survive. *Metabolomics*10.1007/s11306-016-0969-x.

[CR66] Magee, M. J., Salindri, A. D., Kyaw, N. T. T., Auld, S. C., Haw, J. S., & Umpierrez, G. E. (2018). Stress hyperglycemia in patients with tuberculosis disease: Epidemiology and clinical implications. *Current Diabetes Reports*, *18*, 71. 10.1007/s11892-018-1036-y 30090969 10.1007/s11892-018-1036-y PMC6309553

[CR67] Mandi, Y., & Vecsei, L. (2012). The kynurenine system and immunoregulation. *Basic Neuroscience Genetics and Immunology*, *119*, 197–209. 10.1007/s00702-011-0681-y 10.1007/s00702-011-0681-y 21744051

[CR68] Martin, K. S., Azzolini, M., & Ruas, J. L. (2020). The kynurenine connection: How exercise shifts muscle Tryptophan metabolism and affects energy homeostasis, the immune system, and the brain. *American Journal of Physiology-Cell Physiology*, *318*, 818–830. 10.1152/ajpcell.00580.2019 10.1152/ajpcell.00580.2019 32208989

[CR69] Menzies, N. A., Wolf, E., Connors, D., Bellerose, M., Sbarra, A. N., Cohen, T., Hill, A. N., Yaesoubi, R., Galer, K., White, P. J., Abubakar, I., & Salomon, J. A. (2018). Progression from latent infection to active disease in dynamic tuberculosis transmission models: A systematic review of the validity of modelling assumptions. *The Lancet Infectious Diseases*, *18*, e228–e238. 10.1016/S1473-3099(18)30134-8 29653698 10.1016/S1473-3099(18)30134-8 PMC6070419

[CR70] Meregildo-Rodriguez, E. D., Asmat-Rubio, M. G., Zavaleta-Alaya, P., & Vásquez-Tirado, G. A. (2022). Effect of oral antidiabetic drugs on tuberculosis risk and treatment outcomes: Systematic review and Meta-Analysis. *Tropical Medicine and Infectious Disease*, *7*, 343. 10.3390/tropicalmed7110343 36355885 10.3390/tropicalmed7110343 PMC9694577

[CR71] Mingrone, G., Castagneto-Gissey, L., & Mace, K. (2013). Use of Dicarboxylic acids in type 2 diabetes. *British Journal of Clinical Pharmacology*, *75*, 671–676. 10.1111/j.1365-2125.2012.04177.x 22242741 10.1111/j.1365-2125.2012.04177.x PMC3575934

[CR72] Murabayashi, M., Daimon, M., Murakami, H., Kamba, A., & Mizushiri, S. (2018). Association of urinary normetanephrine levels with increased insulin resistance in a general population. *Diabetes*. 10.2337/db18-1655-P

[CR73] Navarro-González, J., & Mora-Fernández, C. (2008). The role of inflammatory cytokines in diabetic nephropathy. *Journal of the American Society of Nephrology (JASN)*, *19*, 433–442. 10.1681/ASN.2007091048 18256353 10.1681/ASN.2007091048

[CR74] Negi, S., Pahari, S., Bashir, H., & Agrewala, J. N. (2020). Intestinal microbiota disruption limits the Isoniazid mediated clearance of Mycobacterium tuberculosis in mice. *European Journal of Immunology*, *50*, 1976–1987. 10.1002/eji.202048556 32673409 10.1002/eji.202048556

[CR75] Niazi, A. K., & Kalra, S. (2012). Diabetes and tuberculosis: A review of the role of optimal glycemic control. *Journal of Diabetes & Metabolic Disorders*. 10.1186/2251-6581-11-28 10.1186/2251-6581-11-28 PMC3598170 23497638

[CR76] Ohra-aho, T., Niemi, P., Aura, A. M., Orlandi, M., Poutanen, K., Buchert, J., & Tamminen, T. (2016). Structure of brewer’s spent grain lignin and its interactions with gut microbiota in vitro. *Journal of Agricultural and Food Chemistry*, *64*, 812–820. 10.1021/acs.jafc.5b05535 26751846 10.1021/acs.jafc.5b05535

[CR77] Ou, K., Sarnoski, P., Schneider, K. R., Song, K., Khoo, C., & Gu, L. (2014). Microbial catabolism of procyanidins by human gut microbiota. *Molecular Nutrition and Food Research*, *58*, 2196–2205. 10.1002/mnfr.201400243 25045165 10.1002/mnfr.201400243

[CR78] Pajuelo, D., Gonzalez-Juarbe, N., Tak, U., Sun, J., Orihuela, C. J., & Niederweis, M. (2018). NAD(+) depletion triggers macrophage Necroptosis, a cell death pathway exploited by Mycobacterium tuberculosis. *Cell Rep*, *24*, 429–440. 10.1016/j.celrep.2018.06.042 29996103 10.1016/j.celrep.2018.06.042 PMC6136256

[CR79] Parveen, S., & Bishai, W. R. (2024). Role of glutamine metabolism in tuberculosis pathogenesis: A mini review. *Frontiers in Tuberculosis*. 10.3389/ftubr.2024.1432880 10.3389/ftubr.2024.1432880 PMC13494981 42638765

[CR80] Pasupuleti, V. R., Arigela, C. S., Gan, S. H., Salam, S. K. N., Krishnan, K. T., Rahman, N. A., & Jeffree, M. S. (2020). A Review on Oxidative Stress, Diabetic Complications, and the Roles of Honey Polyphenols. *Oxidative Medicine and Cellular Longevity* 2020, 8878172. 10.1155/2020/8878172 10.1155/2020/8878172 PMC7704201 33299532

[CR81] Peaston, R. T., & Weinkove, C. (2004). Measurement of catecholamines and their metabolites. *Annals of Clinical Biochemistry*, *41*, 17–38. 10.1258/000456304322664663 14713382 10.1258/000456304322664663

[CR82] Peterson, M. C., & Shulman, G. I. (2018). Mechanisms of insulin action and insulin resistance. *Physiological Reviews*, *98*, 2133–2223. 10.1152/physrev.00063.2017 30067154 10.1152/physrev.00063.2017 PMC6170977

[CR83] Philips, P. A., & Ernst, J. D. (2012). Tuberculosis pathogenesis and immunity. *Annual Review of Pathology: Mechanisms of Disease*, *7*, 353–384. 10.1146/annurev-pathol-011811-132458 10.1146/annurev-pathol-011811-132458 22054143

[CR84] Pires, A. S., Tan, V. X., Heng, B., Guillemin, G. J., & Latini, A. (2020). Kynurenine and tetrahydrobiopterin pathways crosstalk in pain hypersensitivity. *Frontiers in Neuroscience*. 10.3389/fnins.2020.00620 32694973 10.3389/fnins.2020.00620 PMC7338796

[CR85] Ronacher, K., Joosten, S. A., Van Crevel, R., Dockrell, H. M., Walzl, G., & Ottenhoff, T. H. M. (2015). Acquired immunodeficiencies and tuberculosis: Focus on HIV/AIDS and diabetes mellitus. *Immonological Reviews*, *264*, 121–137. 10.1111/imr.12257 10.1111/imr.12257 25703556

[CR86] Sane Schepisi, M., Navarra, A., Altet Gomez, M. N., Dudnyk, A., Dyrhol-Riise, A. M., Esteban, J., Giorgetti, P. F., Gualano, G., Guglielmetti, L., Heyckendorf, J., Kaluzhenina, A., Lange, B., Lange, C., Manika, K., Miah, J., Nanovic, Z., Pontali, E., Prego, M. R., Solovic, I., Tiberi, S., Palmieri, F., & Girardi, E. (2018). Burden and characteristics of the comorbidity Tuberculosis—Diabetes in europe: TBnet prevalence survey and Case-Control study. *Open Forum Infectious Diseases*. 10.1093/ofid/ofy337 30697572 10.1093/ofid/ofy337 PMC6330516

[CR87] Sankaranarayanan, R., Kumar, D. R., Patel, J., & Bhat, G. J. (2020). Do aspirin and flavonoids prevent cancer through a common mechanism involving hydroxybenzoic Acids?-The metabolite hypothesis. *Molecules*. 10.3390/molecules25092243 32397626 10.3390/molecules25092243 PMC7249170

[CR88] Sekhar, R. V., McKay, S. V., Patel, S. G., Guthikonda, A. P., Reddy, V. T., Balasubramanyam, A., & Jahoor, F. (2011). Glutathione synthesis is diminished in patients with uncontrolled diabetes and restored by dietary supplementation with cysteine and Glycine. *Diabetes Care*, *34*, 162–167. 10.2337/dc10-1006 20929994 10.2337/dc10-1006 PMC3005481

[CR89] Sharma, M., Sharma, S., Roy, S., Varma, S., & Bose, M. (2007). Pulmonary epithelial cells are a source of interferon-gamma in response to Mycobacterium tuberculosis infection. *Immunology and Cell Biology*, *85*, 229–237. 10.1038/sj.icb.7100037 17310225 10.1038/sj.icb.7100037

[CR90] Sharma, S., Krishnappa, D., Singh, A. D., Sinha, S., Ammini, A. C., & Soneja, M. (2019). Impact of tuberculosis on glycaemic status: A neglected association. *Indian Journal of Medical Research*. 10.4103/ijmr.IJMR_1927_17 31249204 10.4103/ijmr.IJMR_1927_17 PMC6607812

[CR91] Shastri, M. D., Shukla, S. D., Chong, W. C., Dua, K., Peterson, G. M., Patel, R. P., Hansbro, P. M., Eri, R., O’Toole, R. F., & De Miranda, A. S. (2018). Role of oxidative stress in the pathology and management of human tuberculosis. *Oxidative Medicine and Cellular Longevity*. 10.1155/2018/7695364 30405878 10.1155/2018/7695364 PMC6201333

[CR92] Shibata, K., & Sakamoto, M. (2016). Urinary branched-chain 2-oxo acids as a biomarker for function of B-group vitamins in humans. *Journal of Nutritional Science and Vitaminology*, *62*, 220–228. 10.3177/jnsv.62.220 27725406 10.3177/jnsv.62.220

[CR93] Shillitoe, E., Weinstock, R., Kim, T., Simon, H., Planer, J., Noonan, S., & Cooney, R. (2012). The oral microflora in obesity and type-2 diabetes. *Journal of Oral Microbiology*. 10.3402/jom.v4i0.19013 23119124 10.3402/jom.v4i0.19013 PMC3485401

[CR94] Siddik, M. A. D., & Shin, A. C. (2019). Recent progress on Branched-Chain amino acids in Obesity, Diabetes, and beyond. *Endocrinology and Metabolism*. 10.3803/EnM.2019.34.3.234 31565875 10.3803/EnM.2019.34.3.234 PMC6769348

[CR95] Singhal, A., & Cheng, C. Y. (2018). Host NAD + metabolism and infections: Therapeutic implications. *International Immunology*, *31*, 59–67. 10.1093/intimm/dxy068 10.1093/intimm/dxy068 30329059

[CR96] Storla, D. G., Yimer, S., & Bjune, G. A. (2008). A systematic review of delay in the diagnosis and treatment of tuberculosis. *Bmc Public Health*, *8*, 15. 10.1186/1471-2458-8-15 18194573 10.1186/1471-2458-8-15 PMC2265684

[CR97] Sultana, M. R., Bagul, P. K., Katare, P. B., Anwar Mohammed, S., Padiya, R., & Banerjee, S. K. (2016). Garlic activates SIRT-3 to prevent cardiac oxidative stress and mitochondrial dysfunction in diabetes. *Life Sciences*, *164*, 42–51. 10.1016/j.lfs.2016.08.030 27590611 10.1016/j.lfs.2016.08.030

[CR98] Sun, J., Siroy, A., Lokareddy, R. K., Speer, A., Doornbos, K. S., Cingolani, G., & Niederweis, M. (2015). The tuberculosis necrotizing toxin kills macrophages by hydrolyzing NAD. *Nature Structural & Molecular Biology*, *22*, 672–678. 10.1038/nsmb.3064 10.1038/nsmb.3064 PMC4560639 26237511

[CR99] Suzuki, Y., Suda, T., Asada, K., Miwa, S., Suzuki, M., Fujie, M., Furuhashi, K., Nakamura, Y., Inui, N., Shirai, T., Hayakawa, H., Nakamura, H., & Chidaa, K. (2012). Serum indoleamine 2,3-dioxygenase cctivity predicts prognosis of pulmonary tuberculosis. *Clinical and Vaccine Immunology*, *19*, 436–442. 10.1128/CVI.05402-11 22219312 10.1128/CVI.05402-11 PMC3294601

[CR100] Szymańska, E., Saccenti, E., Smilde, A. K., & Westerhuis, J. A. (2012). Double-check: Validation of diagnostic statistics for PLS-DA models in metabolomics studies. *Metabolomics*, *8*, 3–16. 10.1007/s11306-011-0330-3 22593721 10.1007/s11306-011-0330-3 PMC3337399

[CR101] Theron, I. J., Mason, S., van Reenen, M., Stander, Z., Kleynhans, L., Ronacher, K., & Loots, D. T. (2024). Characterizing poorly controlled type 2 diabetes using (1)H-NMR metabolomics. *Metabolomics*, *20*, 54. 10.1007/s11306-024-02127-w 38734832 10.1007/s11306-024-02127-w PMC11088559

[CR102] Villela, A. D., Sánchez-Quitian, Z. A., Ducati, G., Santos, D. S., & Basso, L. A. (2011). Pyrimidine salvage pathway in Mycobacterium tuberculosis. *Current Medicinal Chemistry*, *18*, 1286–1298. 10.2174/092986711795029555 21366534 10.2174/092986711795029555

[CR103] Vrieling, F., Alisjahbana, B., Sahiratmadja, E., van Crevel, R., Harms, A. C., Hankemeier, T., Ottenhoff, T. H. M., & Joosten, S. A. (2019). Plasma metabolomics in tuberculosis patients with and without concurrent type 2 diabetes at diagnosis and during antibiotic treatment. *Scientific Reports*, *9*, 18669. 10.1038/s41598-019-54983-5 31822686 10.1038/s41598-019-54983-5 PMC6904442

[CR104] Wang, B., Ma, Y., Kong, X., Ding, X., Gu, H., Chu, T., & Ying, W. (2014). NAD(+) administration decreases doxorubicin-induced liver damage of mice by enhancing antioxidation capacity and decreasing DNA damage. *Chem Biol Interact*, *212*, 65–71. 10.1016/j.cbi.2014.01.013 24491677 10.1016/j.cbi.2014.01.013

[CR106] Wang, M., Tan, Y., Shi, Y., Wang, X., Liao, Z., & Wei, P. (2020). Diabetes and sarcopenic obesity: pathogenesis, diagnosis, and treatments. *Frontiers in Endocrinology*. 10.3389/fendo.2020.00568 32982969 10.3389/fendo.2020.00568 PMC7477770

[CR105] Wang, C. Y., Liao, K. M., Wang, Y. H., Chen, K. H., Chuang, S., Liu, C. J., Shu, C. C., & Wang, H. C. (2023). Dipeptidyl peptidase IV inhibitors and the risk of mycobacterial pulmonary infections in type 2 diabetes mellitus. *Journal of Infection and Public Health*, *16*, 1709–1715. 10.1016/j.jiph.2023.08.018 37729686 10.1016/j.jiph.2023.08.018

[CR107] Wilk, A., Hayat, F., Cunningham, R., Li, J., Garavaglia, S., Zamani, L., Ferraris, D. M., Sykora, P., Andrews, J., Clark, J., Davis, A., Chaloin, L., Rizzi, M., Migaud, M., & Sobol, R. W. (2020). Extracellular NAD + enhances PARP-dependent DNA repair capacity independently of CD73 activity. *Scientific Reports*, *10*, 651. 10.1038/s41598-020-57506-9 31959836 10.1038/s41598-020-57506-9 PMC6971268

[CR108] Williamson, J. R., Chang, K., Frangos, M., Hasan, K. S., Ido, Y., Kawamura, T., Nyengaard, J. R., van den Enden, M., Kilo, C., & Tilton, R. G. (1993). Hyperglycemic pseudohypoxia and diabetic complications. *Diabetes*, *42*, 801–813. 10.2337/diab.42.6.801 8495803 10.2337/diab.42.6.801

[CR109] Workneh, M. H., Bjune, G. A., & Yimer, S. A. (2017). Prevalence and associated factors of tuberculosis and diabetes mellitus comorbidity: A systematic review. *PLOS ONE*, *12*, e0175925. 10.1371/journal.pone.0175925 28430796 10.1371/journal.pone.0175925 PMC5400500

[CR110] World Health Organisation (2024). Global tuberculosis report 2024. https://www.who.int/teams/global-tuberculosis-programme/tb-reports

[CR111] Wu, S. H., D., D. and, & Wang, L. C. (2008). A Study of Outlier Analysis Techniques for Delay Testing. *IEEE International Test Conference*. 10.1109/TEST.2008.4700548

[CR112] Xie, N., Zhang, L., Gao, W., Huang, C., Huber, P. E., Zhou, X., Li, C., Shen, G., & Zou, B. (2020). NAD(+) metabolism: Pathophysiologic mechanisms and therapeutic potential. *Signal Transduction and Targeted Therapy*, *5*, 227. 10.1038/s41392-020-00311-7 33028824 10.1038/s41392-020-00311-7 PMC7539288

[CR113] Yen, N. T. H., Anh, N. K., Jayanti, R. P., Phat, N. K., Vu, D. H., Ghim, J. L., Ahn, S., Shin, J. G., Oh, J. Y., Long, N. P., & Kim, D. H. (2023). Multimodal plasma metabolomics and lipidomics in elucidating metabolic perturbations in tuberculosis patients with concurrent type 2 diabetes. *Biochimie*, *211*, 153–163. 10.1016/j.biochi.2023.04.009 37062470 10.1016/j.biochi.2023.04.009

[CR114] Yorke, E., Atiase, Y., Akpalu, J., Sarfo-Kantanka, O., Boima, V., & Dey, I. D. (2017). The bidirectional relationship between tuberculosis and diabetes. *Tuberc Res Treat*, *2017*. 10.1155/2017/1702578 10.1155/2017/1702578 PMC5705893 29270319

[CR118] Zhang, M., & He, J. Q. (2020). Impacts of Metformin on tuberculosis incidence and clinical outcomes in patients with diabetes: A systematic review and meta-analysis. *European Journal of Clinical Pharmacololgy*, *76*, 149–159. 10.1007/s00228-019-02786-y 10.1007/s00228-019-02786-y 31786617

[CR117] Zhang, L. Q., Heruth, D. P., & Ye, S. Q. (2011). Nicotinamide phosphoribosyltransferase in human diseases. *Journal of Bioanalysis and Biomedicine*, *3*, 13–25. 10.4172/1948-593X.1000038 22140607 10.4172/1948-593X.1000038 PMC3227030

[CR116] Zhang, A., Sun, H., Wu, X., & Wang, X. (2012). Urine metabolomics. *Clinica Chimica Acta*, *414*, 65–69. 10.1016/j.cca.2012.08.016 10.1016/j.cca.2012.08.016 22971357

[CR115] Zhang, A., Qiu, S., Xu, H., Sun, H., & Wang, X. (2014). Metabolomics in diabetes. *Clinica Chimica Acta*, *429*, 106–110. 10.1016/j.cca.2013.11.037 10.1016/j.cca.2013.11.037 24321733

[CR119] Zhao, M., Wang, Y., Li, L., Liu, S., Wang, C., Yuan, Y., Yang, G., Chen, Y., Cheng, J., Lu, Y., & Liu, J. (2021). Mitochondrial ROS promote mitochondrial dysfunction and inflammation in ischemic acute kidney injury by disrupting TFAM-mediated MtDNA maintenance. *Theranostics*, *11*, 1845–1863. 10.7150/thno.50905 33408785 10.7150/thno.50905 PMC7778599

[CR120] Zhong, H. J., Yuan, Y., Xie, W. R., Chen, M. H., & He, X. X. (2016). Type 2 diabetes mellitus is associated with more serious small intestinal mucosal injuries. *Plos One*, *11*, e0162354. 10.1371/journal.pone.0162354 27598308 10.1371/journal.pone.0162354 PMC5012602

