# Supplementary material for: Characterizing the tuberculosis and type 2 diabetes mellitus comorbidity in a South African cohort using untargeted GCxGC-TOFMS metabolomics
Source: Metabolomics. 2026 Jan 19;22(1):19. doi: 10.1007/s11306-025-02389-y (PMC12815989; doi:10.1007/s11306-025-02389-y)
Supplement: Supplementary file 1 — Supplementary Material 1 [file 11306_2025_2389_MOESM1_ESM.docx]

**Supplementary information**


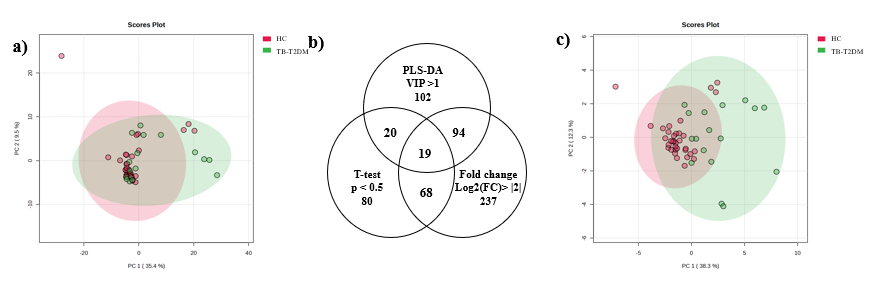


S1 Figure 1: (a) PCA plot illustrates no natural differentiation between TB-T2DM group and HC metabolic profiles, using all samples and metabolites. (b) A Venn diagram, illustrating the number of metabolites meeting each criterium and their overlaps of common metabolites forming the noise-reduced data set. (c) PCA plot of noise-reduced dataset illustrates separation between the TB-T2DM and the HC group.
